# mRNA analysis identifies deep intronic variants causing Alport syndrome and overcomes the problem of negative results of exome sequencing

**DOI:** 10.1038/s41598-021-97414-0

**Published:** 2021-09-10

**Authors:** Xiaoyuan Wang, Yanqin Zhang, Jie Ding, Fang Wang

**Affiliations:** Department of Pediatrics, Peking University First Hospital, Beijing, 100034 China

**Keywords:** Genetics, Molecular medicine, Nephrology

## Abstract

Mutations in *COL4A3*, *COL4A4* and *COL4A5* genes lead to Alport syndrome (AS). However, pathogenic variants in some AS patients are not detected by exome sequencing. The aim of this study was to identify the underlying genetic causes of five unrelated AS probands with negative next-generation sequencing (NGS) test results. Urine *COL4A3–5* mRNAs were analyzed in the probands with an uncertain inherited mode of AS, and *COL4A5* mRNA of skin fibroblasts was analyzed in the probands with X-linked AS. RT-PCR and direct sequencing were performed to detect mRNA abnormalities. PCR and direct sequencing were used to analyze the exons with flanking intronic sequences corresponding to mRNA abnormalities. Six novel deep intronic splicing variants in *COL4A4* and *COL4A5* genes that cannot be captured by exome sequencing were identified in the four AS probands. Skipping of an exon was caused by an intronic variant, and retention of an intron fragment caused by five variants. In the remaining AS proband, *COL4A5* variants c.2677 + 646 C > T and r.2678_r.2767del were detected at the DNA and RNA level, respectively, whereas it is unclear whether c.2677 + 646 C > T may not lead to r.2678_r.2767del. Our results reveal that mRNA analysis for AS genes from either urine or skin fibroblasts can resolve genetic diagnosis in AS patients with negative NGS results. We recommend analyzing *COL4A3–5* mRNA from urine as the first choice for these patients because it is feasible and non-invasive.

## Introduction

Alport syndrome (AS) is a hereditary nephritis characterized by hematuria, proteinuria, and progressive renal failure and is sometimes accompanied by sensorineural deafness and ocular abnormal^1^. The three genetic forms of Alport syndrome depend on the mode of inheritance: X-linked AS (XLAS), autosomal recessive AS (ARAS), and autosomal dominant AS (ADAS)^2,3^. XLAS is caused by pathogenic variants in the *COL4A5* gene, while ARAS and ADAS are caused by pathogenic variants in the *COL4A3* or *COL4A4* gene^4^. Pathogenic variants in *COL4A3*, *COL4A4* or *COL4A5* genes lead to abnormal α3, α4 or α5 chains of type IV collagen in the glomerular basement membrane (GBM). The gold standard for clinical diagnosis of AS is the characteristic changes in GBM, including irregular thickening, splitting and “basket-weave” changes, seen under an electron microscope^5^. Genetic testing for pathogenic variants in *COL4A3*, *COL4A4*, or *COL4A5* genes is currently used frequently in the diagnosis of AS because of the increasing availability of next-generation sequencing (NGS), including targeted NGS and whole exome sequencing, in the clinic^6^. NGS detects approximately 82%–86% of pathogenic *COL4A3–5* gene variants^6,7^. However, some genetic changes that cause AS, such as deep intronic splicing variants, somatic mosaicism, and copy number variants, are not detectable by NGS^8–10^.

mRNA sequencing is an effective method to identify intronic splicing variants. Several *COL4A5* gene deep intronic splicing variants have been reported in studies that analyzed mRNA from skin fibroblasts^11^, peripheral blood lymphocytes^12^, hair root^13^, or renal tissue^14^. In our clinical practice, since 2000, simultaneous examinations of α5(IV) staining in skin and *COL4A5* mutation screening using mRNA extracted from cultured skin fibroblasts have been routinely performed in patients with suspected XLAS. However, this approach cannot be applied for Alport syndrome patients with autosomal inherited patterns, since α3(IV) and α4(IV) are not expressed in skin. Two deep intronic variants in the *COL4A3* gene were identified by analysis of mRNA from blood or urine^15^. However, to our knowledge, the value of detecting mutations in *COL4A5* and *COL4A4* genes in mRNA isolated from urine has not been adequately studied.

The aim of this study was to identify the genetic etiologies of five unrelated AS patients with negative NGS results. We used our developed approach for analysis of the entire coding regions of *COL4A3*, *COL4A4*, and *COL4A*5 mRNAs isolated from urine and *COL4A5* mRNA extracted from cultured skin fibroblasts and identified deep intronic splicing variants in the enrolled patients. These findings indicate that our developed approach may help guide medical practitioners and genetic counselors to provide personalized management of AS.

## Results

### Analysis of urine NPHS2 and COL4A3-5 mRNAs of the control

**Figure 1:**
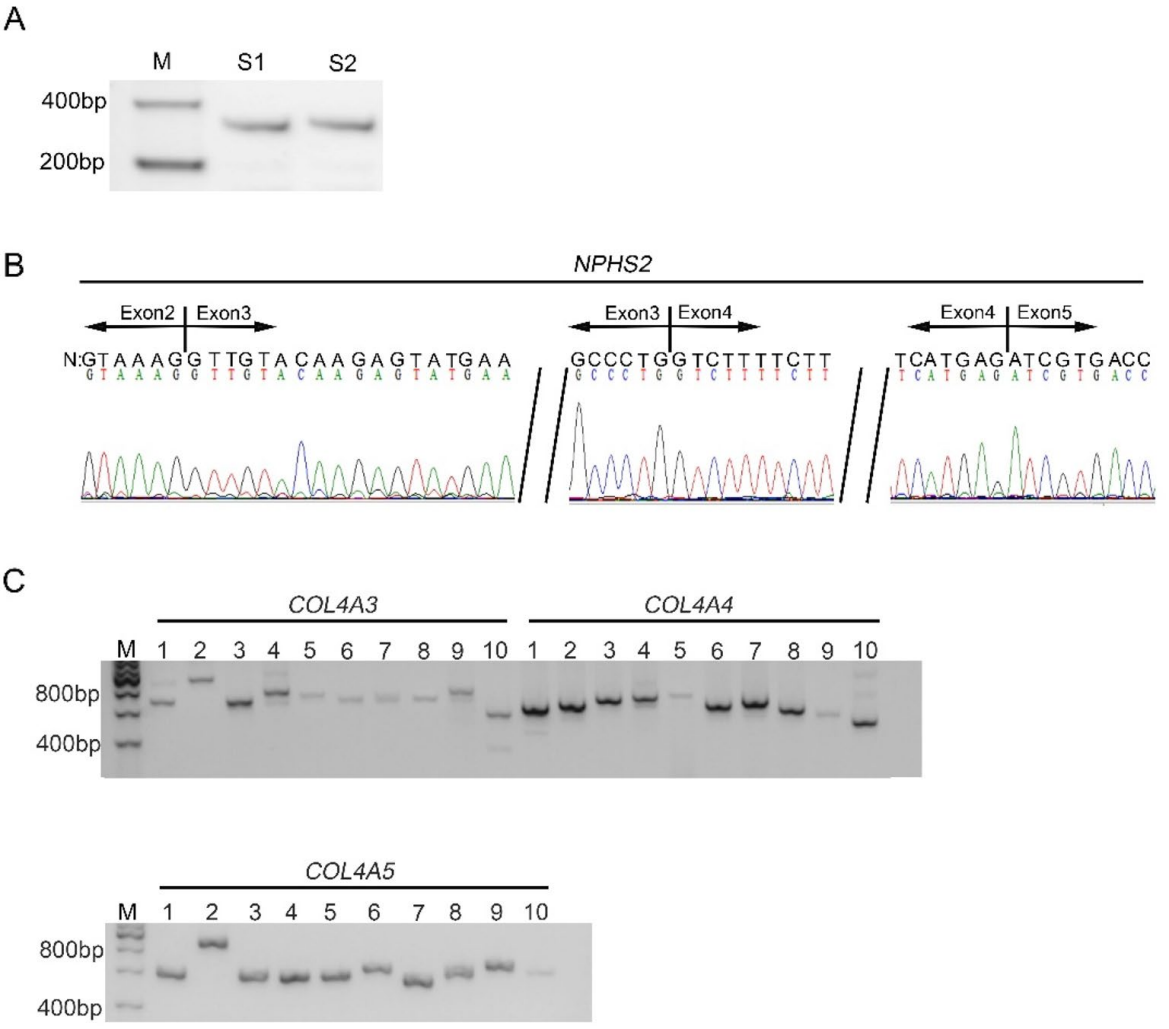
Agarose gel electrophoresis showed urine *NPHS2* and *COL4A3-5* mRNAs of the control. (**A**) and (**B**) showed agarose gel electrophoresis and sequences of the products of two independent RT-PCR assays for *NPHS2* mRNA transcript in the control’s urine; (**C**) showed agarose gel electrophoresis of *COL4A3*, *COL4A4*, or *COL4A5* mRNA. M: DNA molecular mass marker. S1and S2: the products of two independent RT-PCR tests for NPHS2 mRNA. Lane 1 to 10: 10 overlapping PCR products covering the entire selected gene cDNA from the control.

As show in Fig. 1A,B, agarose gel electrophoresis showed that the sizes of the products of two independent RT-PCR assays for *NPHS2* mRNA transcript in the control’s urine were the same as initially designed, and subsequent sequencing demonstrated the amplified sequence corresponded exactly to the 388 bp of the published *NPHS2* mRNA sequence (NM_014625.3), which indicated that urine pellets contain podocytes. The sizes of all ten overlapping fragments covering the entire coding sequence of either *COL4A3*, *COL4A4*, or *COL4A5* mRNA were the same as originally conceived (Fig. 1C), and sequencing of these RT–PCR products confirmed that the amplified sequences mapped precisely to the published *COL4A3*, *COL4A4*, and *COL4A5* mRNA sequences (NM_000091.5, NM_000092.5, NM_000495.5 and NM_033381), respectively. The first author can provide the original data if needed.

### Clinical features of AS patients

**Figure 2:**
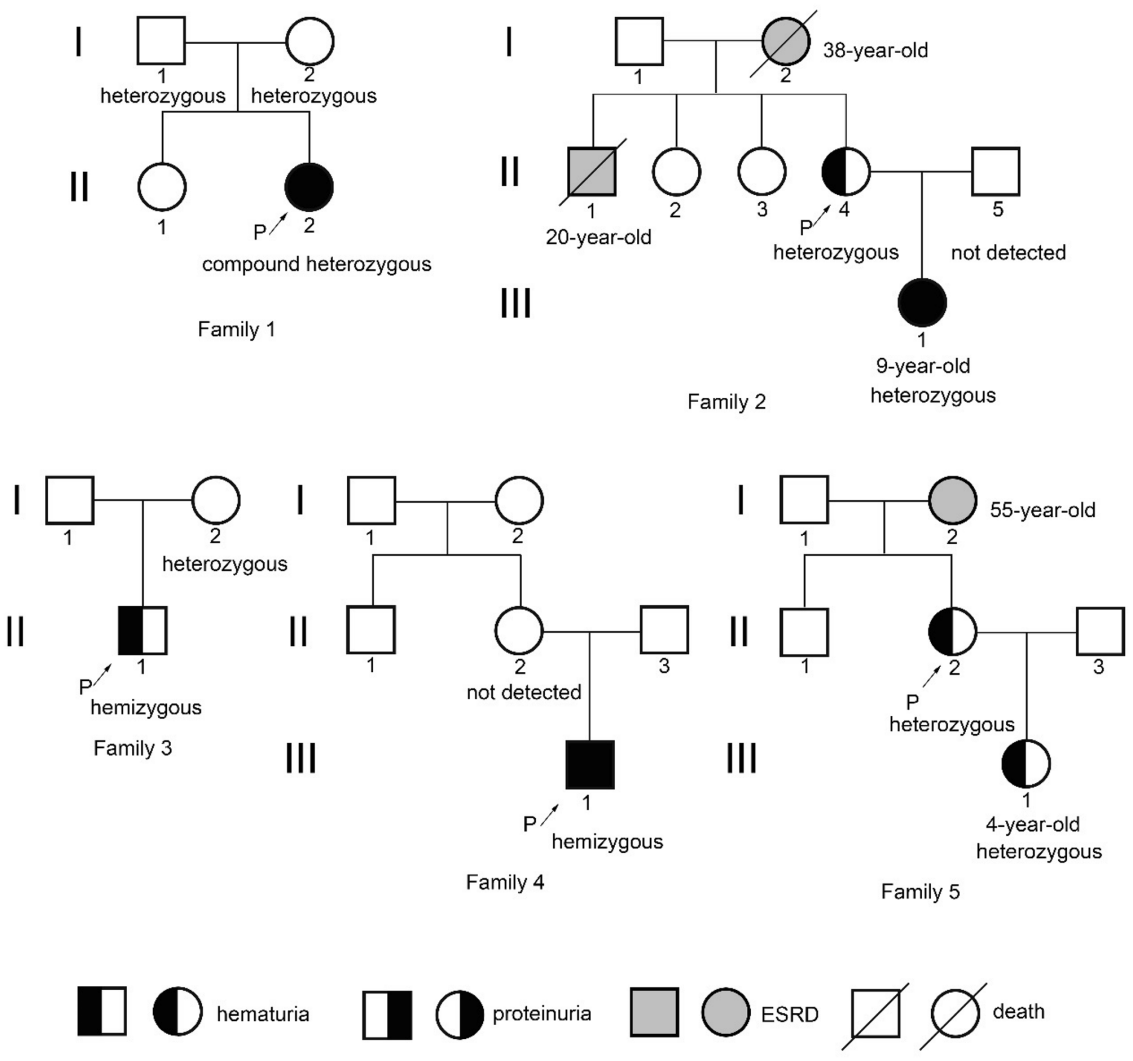
Pedigrees of the 5 families in this study. The proband is indicated by an arrow. The status of disease-causing variants is indicated below the individuals undergoing segregation analyses. Patient III-1 of family 2 presented with proteinuria of 0.534 g/24 h at the age of 7 years.

Five unrelated Alport syndrome probands were enrolled according to the inclusion and exclusion criteria listed in the Material and Methods. Patient clinical information and pedigrees are shown in Table 1 and Fig. 2, respectively. Proband 1 was diagnosed with AS based on characteristic AS features in GBM; the inheritance pattern was uncertain because of a negative family history and normal staining of α5(IV) chain in skin tissue. Proband 2 was highly suspected of having XLAS based on a positive family history of hematuria and end stage renal disease (ESRD) and diffuse thinning of the GBM (less than 200 nm shown by ultrastructural examination of 3 glomeruli) found in her daughter’s renal biopsy taken at age 3.25 years in another hospital; however, normal staining of α5(IV) chain in skin tissue did not support the diagnosis. XLAS was diagnosed in probands 3–5 with abnormal staining of α5(IV) chain in skin specimens.

**Table 1:** Clinical features and analyzed samples of the 5 probands in this study.

Proband	Gender	Age of onset (y)	Hematuria	Proteinuria	Serum creatinine* (umol/L)	Family history**	Renal EM	Alpha 5 (IV) in skin EBM	Mode of inheritance	Skin mRNA	Urine mRNA	Blood DNA
1	F	25	+	+	91	−	AS	Positive	Uncertain	−	+	+
2	F	30	+	−	66.71	+	ND	Positive	Uncertain	−	+	+
3	M	3	+	−	30.9	+	ND	Negative	X-linked	+	−	+
4	M	6.8	+	+	78	−	AS	Negative	X-linked	+	−	+
5	F	33	+	−	NA	+	ND	Segmental positive	X-linked	+	−	+

Patients 1 and 4 did not undergo staining of type IV collagen α5 chain in their renal specimen; hearing loss and ocular changes were not detected in five patients.

*Age at which serum creatinine tests were carried out in the four probands was 25, 30, 10 and 14 years, respectively.

**Positive family history of hematuria and/or end stage renal disease.

*EM* electron microscopy, *EBM* epithelial basement membrane, *NA* not available, *ND* not done.

### Gene variants in the five probands

**Figure 3:**
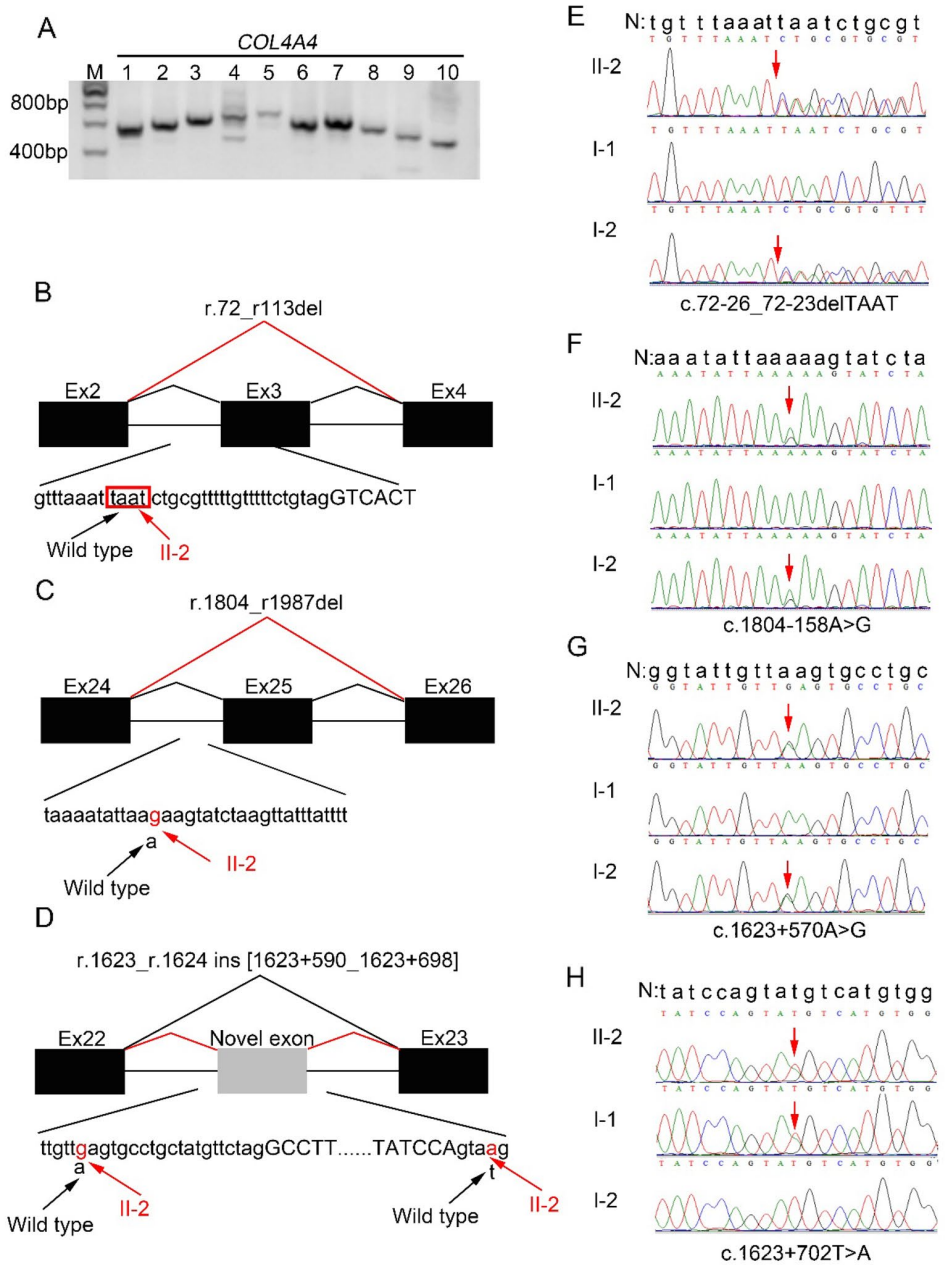
*COL4A4* gene analysis in proband 1 (II-1 of family 1). (**A**) Agarose-gel images of RT-PCR products for proband 1 (II-1 of family 1) urine. M: DNA molecular mass marker. Lane 1 to 10: 10 overlapping PCR products covering the entire *COL4A4* cDNA from proband 1 (II-1 of family 1). (**B**, **C**, and **D**) Schematic representation of the aberrant *COL4A4* cDNA caused by the deep intronic splice variants detected in proband 1 (II-1 of family 1). Exons are represented by black filled rectangles and are numbered, and the novel exon is indicated by the gray filled rectangle. Capital and lowercase letters depict exon and intron sequences, respectively. The black and red lines indicate wild-type and abnormal splicing, respectively. The black arrows indicate the wild-type nucleotide(s), the red arrows show the changed nucleotides, and the red box shows the nucleotides that should have been detected in wild-type but were deleted in the proband. (**E**, **F**, **G**, and **H**) Sequencing of PCR amplified products containing the deep intronic variants for family 1. N, wild type sequence. Intron sequences are depicted by lowercase letters. The red arrows indicate the breakpoint and changed nucleotide.

In proband 1 (II-2 of family 1), cDNA analysis showed that no abnormal transcripts were detected in *COL4A3* and *COL4A5* mRNAs isolated from proband 1’s urine (Suppl. Figure 1A and B). Agarose gel electrophoresis revealed *COL4A4* mRNA transcript in proband 1’s urine was successfully amplified (Fig. 3A). Sequencing of 10 RT-PCR products revealed a heterozygous skipping of exons 3 and 25 (r.72_r113del, p.Trp24*; r.1804_r1987del, p.Gly602Valfs*8; Fig. 3B,D, Suppl. Figure 2A and C) and an insertion of a 109 bp sequence of intron 22 between exons 22 and exon 23 (r.1623_r.1624 ins [1623 + 590_1623 + 698], p.Gly542Alafs*29; Fig. 3C, Suppl. Figure 2B). To further confirm the anomalies detected at the cDNA level, *COL4A4* exons 2–4, 22, and 24–26 with the sequences of flanking introns 2–3, 22, and 24–25 were amplified by PCR from genomic DNA of proband 1 and her parents. Sequence analysis demonstrated that proband 1 and her mother (I-2) were heterozygous for the variants in intron 2 c.72-26_72-23delTAAT, intron 22 c.1623 + 570A > G, and intron 24 c.1804–158A > G (Fig. 3E, F, and G). Proband 1 and her father were heterozygous for the variant in intron 22 c.1623 + 702 T > A (Fig. 3H). Of these four variants, variants c.72-26_72-23delTAAT and c.1623 + 702 T > A had not been documented in gnomAD, Human Gene Mutation Database (HGMD) and ClinVar, whereas the frequency of variants c.1623 + 570A > G and c.1804–158A > G in gnomAD is 6.74% and 62.97%, respectively. These data indicated that the former two variants led to aberrant splicing of exon 3 and intron 22, and the latter two variants are benign variants. *COL4A4* mRNA from urine of proband 1’s father was further analyzed (urine from proband 1’s mother was not available). Sequencing of the fragment 4 RT-PCR product showed that the father (I-1) also had a heterozygous insertion of a 109 bp sequence of intron 22 between exons 22 and exon 23 (r.1623_r.1624 ins [1623 + 590_1623 + 698], p.Gly542Alafs*29) (data not shown). To detect the variant leading to skipping of exon 25, the sequences including introns 23 and 24, exons 24, 25 and 26, and a part of intron 25 were analyzed. No sequence alterations were found. Due to the sequence complexity of intron 25, a fragment of 1.5 kb could not be successfully amplified, we speculated that the pathogenic variant might be located in this fragment.

**Figure 4:**
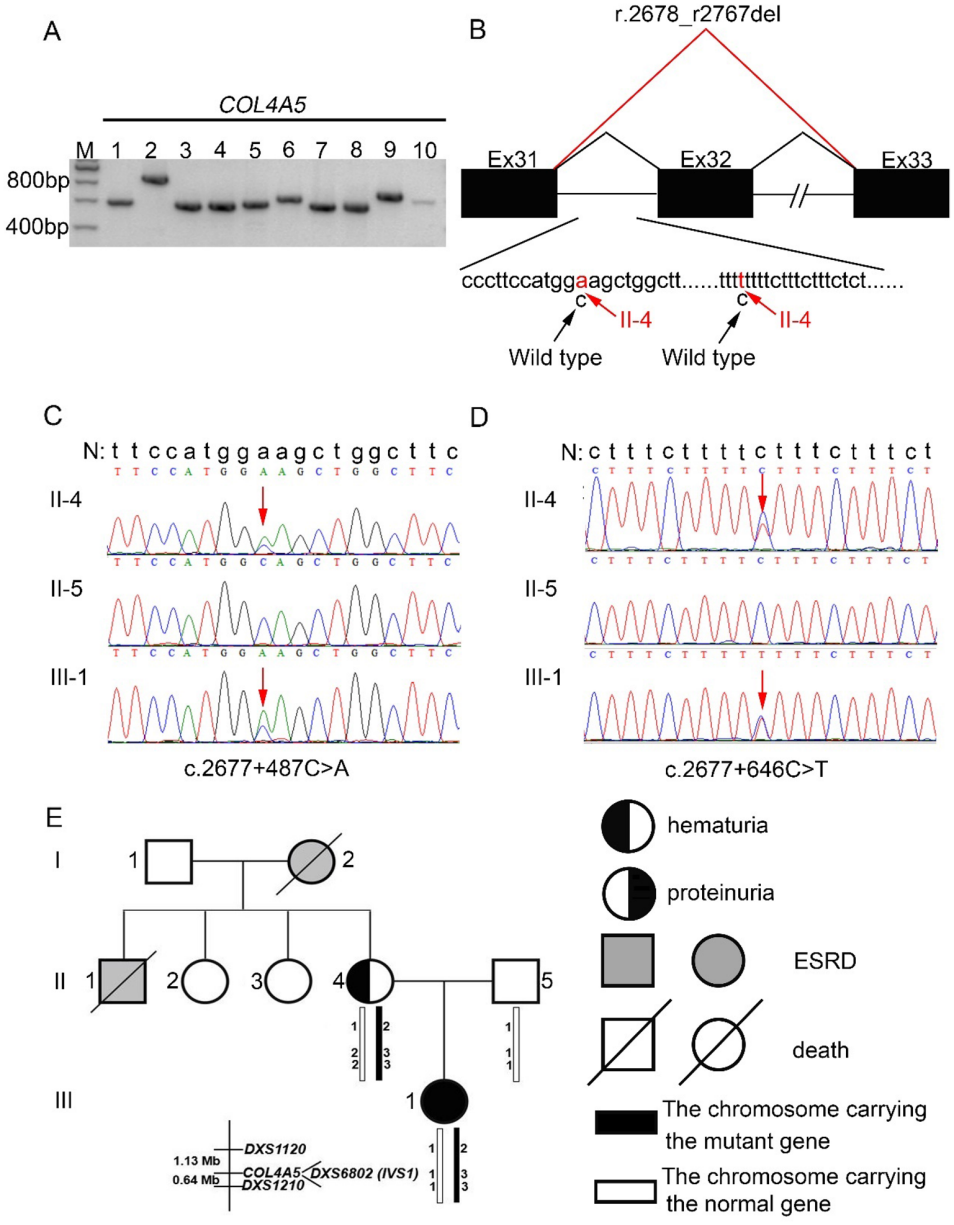
*COL4A5* gene analysis in proband 2 (II-4 of family 2) and haplotypes analysis in her family. (**A**) Agarose gel images of RT-PCR products for proband 2 (II-4 of family 2) urine. M: DNA molecular mass marker. Lane 1 to 10: 10 overlapping PCR products covering the entire *COL4A5* cDNA. (**B**) Schematic representation of the aberrant *COL4A5* cDNA caused by the deep intronic splice variants detected in proband 2 (II-4 of family 2). Exons are represented by black filled rectangles and are numbered. Lowercase letters are used to depict intron sequences. The black and red lines indicate wild-type and abnormal splicing, respectively. The black arrows indicate the wild-type nucleotide(s), the red arrows show the changed nucleotide. (**C** and **D**) Sequencing of PCR amplified products containing the deep intronic variants for family 2. N, wild type sequence. Intron sequences are depicted by lowercase letters. The red arrows indicate the changed nucleotide. (**E**) Linkage analysis using short tandem repeats around the *COL4A5* gene. Haplotypes are demonstrated below each symbol.

In proband 2 (II-4 of family 2), cDNA analysis showed that no abnormal transcripts were detected in *COL4A3* and *COL4A4* mRNAs isolated from proband 2’s urine (Suppl. Figure 1C and D). Agarose gel electrophoresis revealed *COL4A5* mRNA transcript in proband 2’s urine was successfully amplified (Fig. 4A). Sequencing of 10 RT-PCR products revealed that exon 32 of *COL4A5* gene was skipped heterozygously (r.2678_r2767del, Suppl. Figure 2D), which led to an in-frame deletion (p.Thr894_Gly923del) (Fig. 4B). *COL4A5* exons 31–33 with the sequences of the flanking introns 31–32 were amplified by PCR from genomic DNA for proband 2, her husband, and her daughter and then sequenced. Proband 2 and her daughter (III-1) were heterozygous for the variant intron 31 c.2677 + 487C > A and c.2677 + 646C > T (Fig. 4C,D). Neither of the two variants were identified in her husband (II-5). The frequency of variant c.2677 + 487 C > A in gnomAD is 21.96%, that means it is a benign variant. The variant c.2677 + 646 C > T had not been reported in gnomAD, HGMD and ClinVar. In addition, haplotype reconstruction demonstrated an X-linked inheritance mode in family 2 (Fig. 4E).

**Figure 5:**
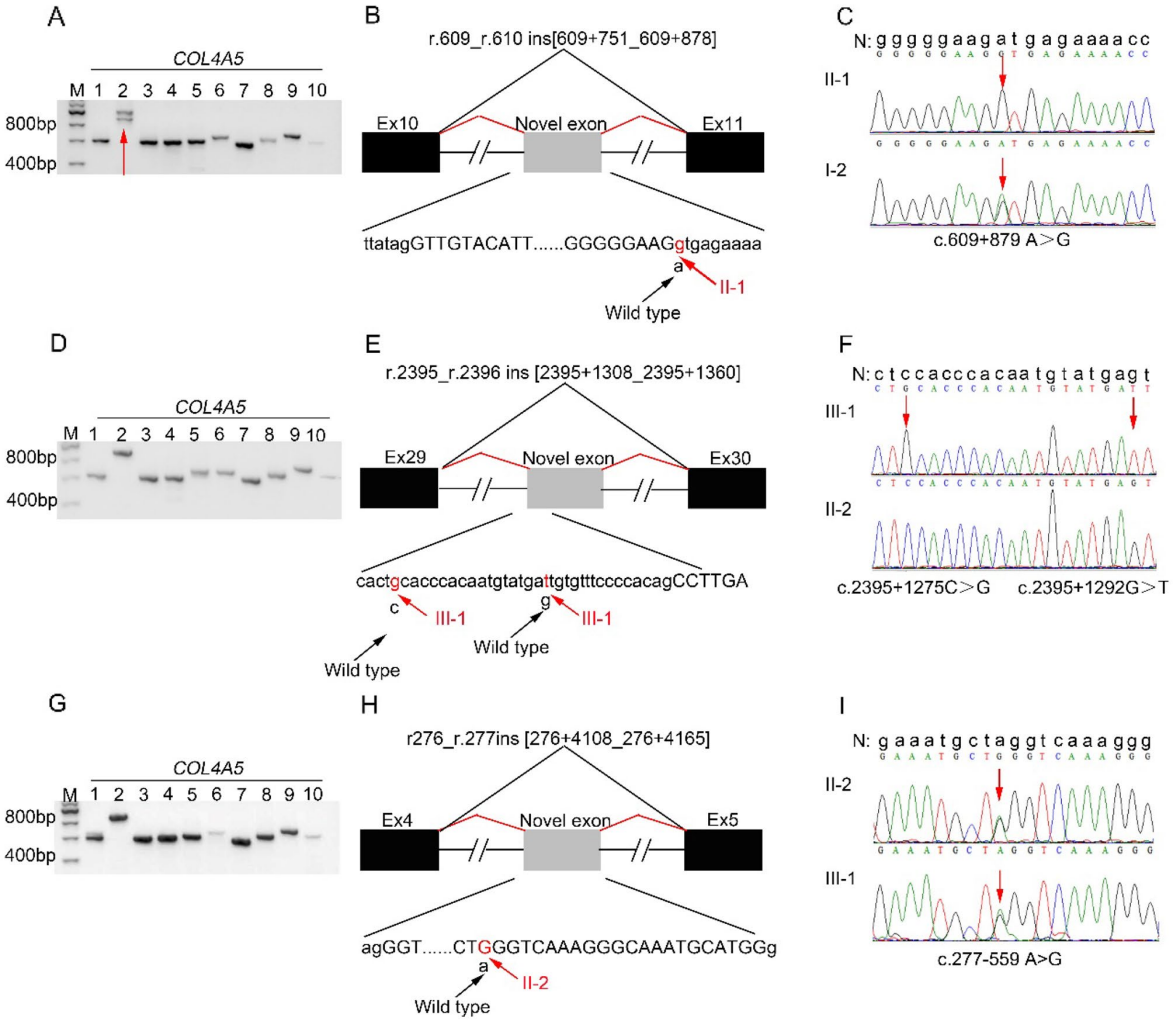
*COL4A5* gene analysis in probands 3–5 (II-1 of family 3, III-1 of family 4, and II-2 of family 5). (**A**, **D**, and **G**) Agarose gel images of RT-PCR products for skin fibroblasts of probands 3–5 (II-1 of family 3, III-1 of family 4, and II-2 of family 5), respectively. M: DNA molecular mass marker. Lane 1 to 10: 10 overlapping PCR products covering the entire *COL4A5* cDNA from probands 3–5 (II-1 of family 3, III-1 of family 4, and II-2 of family 5). The red arrow indicates an abnormal transcript in addition to the wild-type transcript. (**B**, **E**, and **H**) Schematic representation of the aberrant *COL4A5* cDNA caused by the deep intronic splice variants detected in probands 3–5 (II-1 of family 3, III-1 of family 4, and II-2 of family 5), respectively. Exons are represented by black filled rectangles and are numbered, and the novel exon is indicated by the gray filled rectangle. Capital and lowercase letters are used to depict exon and intron sequences, respectively. The black and red lines indicate wild-type and abnormal splicing, respectively. The black arrows indicate the wild-type nucleotide, and the red arrows showed the changed nucleotide. (**C**), (**F**), and (**I**): sequencing of PCR amplified products containing the deep intronic variants for families 3–5, respectively. N, wild type sequence. Intron sequences are depicted by lowercase letters. The red arrows indicate the changed nucleotide.

In proband 3 (II-1 of family 3), agarose gel electrophoresis of RT-PCR fragment 2 products of *COL4A5* mRNA from skin fibroblasts showed an abnormal transcript in addition to the wild-type transcript (Fig. 5A). Sequencing of 10 RT-PCR products revealed that a 128 bp sequence from intron 10 was inserted between exon 10 and exon 11 (r.609_r.610 ins[609 + 751_609 + 878]) (Fig. 5B, Suppl. Figure 3A), which led to premature termination of α5(IV) chain (p.Gly204Valfs*7). *COL4A5* intron 10 was amplified by PCR from genomic DNA in proband 3 and his mother (I-2). Sequencing revealed an A to G change in intron 10 at 879 bp downstream from exon 10 (IVS10 c.609 + 879 A > G) (Fig. 5C) in proband 3 and his mother. This variant had not been documented in gnomAD, HGMD and ClinVar.

In proband 4 (III-1 of family 4), agarose gel electrophoresis revealed *COL4A5* mRNA transcript in proband 4’s skin fibroblasts was successfully amplified (Fig. 5D). Sequencing of 10 RT-PCR products revealed a 53 bp sequence from intron 29 of *COL4A5* gene in *COL4A5* mRNA from skin fibroblasts inserted between exons 29 and exon 30 (r.2395_r.2396 ins [2395 + 1308_2395 + 1360]) (Fig. 5E, Suppl. Figure 3B), which led to premature termination of α5(IV) chain (p.Gly799Alafs*15). *COL4A5* intron 29 was amplified by PCR from genomic DNA in proband 4 and his mother (II-2). A hemizygous C to G variant in intron 29 at 1275 bp and a G to T variant in intron 29 at 1292 bp downstream from exon 29 (IVS29 c.2395 + 1275C > G and c.2395 + 1292G > T) were found in genomic DNA of proband 4 (Fig. 5F). Neither of the two variants were identified in his mother. These two variants had not been documented in gnomAD, HGMD and ClinVar.

In proband 5 (II-2 of family 5), agarose gel electrophoresis revealed *COL4A5* mRNA transcript in proband 5’s skin fibroblasts was successfully amplified (Fig. 5G). Sequencing of 10 RT-PCR products revealed a 58 bp sequence from intron 4 of *COL4A5* gene in *COL4A5* mRNA inserted between exon 4 and exon 5 heterozygously (r276_r.277ins [276 + 4108_276 + 4165]) (Fig. 5H, Suppl. Figure 3C), which led to premature termination of α5(IV) chain (p.Pro94Ilefs*7). *COL4A5* intron 4 was amplified by PCR from genomic DNA in proband 5 and his daughter (III-1). A heterozygous A to G variant in intron 4 at 559 bp upstream from exon 5 (IVS4 c.277–559 A > G) was found in genomic DNA of proband 5 and her daughter (Fig. 5I). This variant had not been reported in gnomAD, HGMD and ClinVar.

According to the American College of Medical Genetics and Genomics guidelines^16^, the foregoing six rare deep intronic variants were classified as pathogenic variants, except *COL4A5* variant c.2677 + 646 C > T (Table 2).

**Table 2:** The output of in silico splice tools for deep intronic pathogenic variants detected in this study.

Proband	DNA level (experimentally determined)	RNA level (experimentally determined)	Protein change	Classification following ACMG criteria	In silico splice tools									
					Human Splicing Finder		NNSPLICE		NetGene2		SpliceAI		MaxEntScan	
					Acceptor	Donor	Acceptor	Donor	Acceptor	Donor	Acceptor	Donor	Acceptor	Donor
1	*COL4A4* intron 2: c.72-26_72-23delTAAT	Del exon 3 (r.72_r.113del)	p (Trp24*)	Pathogenic (PVS1 PM2 PP4)	No significant impact on splicing signals		WT:1.00	WT:0.99	WT: 0.26	WT:0.63	Damage		–	
							Mut:1.00	Mut:0.99	Mut: 0.26	Mut:0.67				
	*COL4A4* intron 23: c.1623 + 702 T > A	Retention 109 nucleotide of intron 22 (r.1623_r.1624 ins [1623 + 590_1623 + 698])	p (Gly542Alafs*29)	Pathogenic (PVS1 PM2 PP4)	Significant alteration of ESE / ESS motifs ratio		WT:0.76	WT:0.67	WT: 0	WT:0.41	–			WT:5.8
							Mut:0.76	Mut:0.96	Mut: 0	Mut:0.70				Mut:9.09
2	*COL4A5* intron 31: c.2677 + 646 C > T	Del exon 32 (r.2678_r.2767del)	p (Thr894_Gly923del)	Variant of uncertain significance (PM2 PP1 PP4)	No significant impact on splicing signals		WT:0.79	WT:0.80	WT: 0.79	WT: 0.80	–			No significant impact on splicing signals
							Mut:0.79	Mut:0.80	Mut: 0.79	Mut: 0.80				
3	*COL4A5* intron 10: c.609 + 879A > G	Retention 128 nucleotide of intron 10 (r.609_r.610 ins[609 + 751_609 + 878])	p (Gly204Valfs*7)	Pathogenic (PVS1 PM2 PP4)	–	WT:67.2	WT:0.94	WT: 0	WT:0.43	WT: 0	–			WT:0.5
						Mut:94.34	Mut:0.94	Mut:0.90	Mut:0.43	Mut: 0.41				Mut:8.68
4	*COL4A5* intron 29: c.2395 + 1275C > G	Retention 53 nucleotide of intron 29 (r.2395_r.2396 ins [2395 + 1308_2395 + 1360])	p (Gly799Alafs*15)	Pathogenic (PVS1 PM2 PP4)	Significant alteration of ESE / ESS motifs ratio		WT:0.65	WT:0.99	WT: 0	WT:0.46	–	–		
							Mut:0.65	Mut:0.99	Mut: 0	Mut:0.46				
	*COL4A5* intron 29: c.2395 + 1292G > T	Retention 53 nucleotide of intron 29 (r.2395_r.2396 ins[2395 + 1308_2395 + 1360])	p.(Gly799Alafs*15)	Pathogenic (PVS1 PM2 PP4)	No significant impact on splicing signals		WT:0.65	WT:0.99	WT: 0	WT:0.46	–			No significant impact on splicing signals
							Mut:0.89	Mut:0.99	Mut: 0.26	Mut:0.46				
5	*COL4A5* intron 4:c.277–559 A > G	Retention 58 nucleotide of intron 4 (r.276_r.277ins [276 + 4108_276 + 4165])	p.(Pro94Ilefs*7)	Pathogenic (PVS1 PM2 PP4)	No significant impact on splicing signals		WT:0.87	WT:0.57	WT:0.71	WT: 0	Damage			No significant impact on splicing signals
							Mut:0.87	Mut:0.57	Mut:0.77	Mut: 0				

Variants were named following Human Genome Variation Society guidelines (http://www.hgvs.org/mutnomen). For genomic DNA positioning, numbering was based on the reference sequences (chr2:227,007,325–227,147,483 for *COL4A4* gene and chrX:108,440,126–108,696,378 for *COL4A5* gene), whereas RNA position was numbered according to the reference sequences (NM_000092.5 for *COL4A4* mRNA and NM_000495 for *COL4A5* mRNA) using the first coding ATG of exon 1 as the initiation codon.

*ACMG* the American College of Medical Genetics and Genomics.

*WT* wild-type sequence, *Mut* mutant sequence, *ESE* exonic splicing enhancer, *ESS* exonic splicing silencer.

### In silico prediction of deep intronic pathogenic variants

Table 2 shows the output of HSF, NNSPLICE, NetGene2, SpliceAI, and MaxEntScan for each rare deep intronic splice variant identified in this study. Only two out of the seven variants (*COL4A4*: c.1623 + 702 T > A and *COL4A5:* c.609 + 879A > G) were correctly predicted as deleterious by four tools, 3 variants were predicted by two tools, and one variant was only predicted by one tool. However, in the case of the residue variant *COL4A5: c.2677* + *646 C* > *T*, no effect on RNA splicing was predicted by four tools, whereas skipping of exon 32 was observed at the RNA level.

## Discussion

In this study, by analyzing *COL4A3–5* mRNAs from urine or skin fibroblasts, six deep intronic pathogenic variants were identified in four unrelated AS patients with negative NGS results, although it is difficult to assess the contribution of *COL4A5* variant c.2677 + 646 C > T to aberrant RNA transcript containing variant r.2678_r.2767del detected in the remaining AS patient. These findings indicate that our developed approach may be applied to help provide personalized evaluation and care of patients and their families. Meanwhile, compared with the mRNA-based approach using skin fibroblasts for finding (likely) pathogenic variants leading to XLAS, the method using urine mRNA has a clear advantage to identify the underlying genetic causes of AS with uncertain inheritance pattern. In addition, this is the first report on compound heterozygous deep intronic splicing mutations in *COL4A4* gene in an AS patient.

Numerous studies have shown that NGS is effective in finding single nucleotide variations and small indels in exons and the flanking intronic regions^17^. However, some genetic events such as deep intronic variants, copy number variants, and somatic cell mosaicism may be missed by NGS^18^. Therefore, for a patient with clinically diagnosed or suspected AS and no pathogenic variants detected by NGS, it is necessary to further analyze *COL4A3–5* genes by mRNA sequencing, chromosome microarray analysis, droplet digital PCR or other approaches to improve genetic diagnosis^19,20^.

According to the literature and public databases (HGMD and Leiden Open source DNA Variation Database), pathogenic splicing variants account for 14.9% to 24.5% in the *COL4A5* gene^21,22^. Approximately 70.4% (112/159) occurred at consensus splice sites, and only seven splicing variants occurred in introns at more than 100 base pairs up/downstream from exon–intron junctions. Approximately 70% (23/32) of the pathogenic *COL4A3* splicing variants occurred at consensus splice sites and only two variants were located in introns at more than 100 base pairs upstream from the exons. No deep intronic *COL4A4* splicing variants have been reported to date. These findings indicate that deep intronic *COL4A3–5* mutations are rare. The six novel deep intronic pathogenic variants obtained in the present study extend the mutational spectrum of AS. These findings also highlight *COL4A3–5* mRNA analysis as an effective supplementary approach for NGS in molecular diagnosis of this disease.

Previous studies have reported that GBM collagen α3α4α5(IV) is synthesized solely by podocytes^23^, and the urine podocyte detachment rate (assessed by podocin mRNA in urine pellets) is increased in AS patients^24,25^. Therefore, extraction of RNA directly from patient-originated urine may be a valuable approach to the analysis of all three Alport gene variants, which was demonstrated by the findings of the present study. Previous studies showed urine-derived podocyte-lineage cells could be used as the primary material for identifying the variants in known nephropathy genes^26,27^, whereas compared with this method our developed approach for isolation of RNA directly from urine is simpler and more practical. A weak point of our approach is the requirement for patient cooperation to obtain enough fresh urine, which means that young patients who cannot rapidly drink 1000–1500 ml water are not suitable for urine mRNA analysis. Meanwhile, the complexity of intron sequences may be unfavorable for amplification and sequencing to detect the variants, and compelling evidence is needed to assess the relationship between exon skipping in the causative gene mRNA observed in the patient-originated urine and the only plausible genomic variant in the candidate region. As family 2 presented here, a positive family history of hematuria and ESRD, diffuse thinning of the GBM observed in the proband’s daughter, and the haplotype of three microsatellite markers around the *COL4A5* gene co-segregated with the proband and her affected daughter were important clues in making a diagnosis of XLAS. Whereas the results of limited splicing computational tools did not implicate the only novel rare deep intronic DNA variant in the *COL4A5* gene caused the aberrant splicing pattern we observed. Although the causative gene mature mRNA pseudo-exon inclusion appears to be the major effect of deep intronic DNA variants, exon skipping from intronic DNA variants away from the canonical splice sites had been reported ^28–30^.

Given that the deep intronic variants identified in the present study could be detected using whole genome sequencing, and in silico splicing prediction tools are usually used to select variants that are predicted to have an effect on splicing in a molecular diagnostic setting^31^, we assessed the reliability of HSF, NNSPLICE, NetGene2, SpliceAI, and MaxEntScan in discriminating between neutral and pathogenic variants. Assuming that the splice outcomes obtained from one tool were consistent with transcript analysis results, six variants detected in this study were correctly predicted, which indicated that these tools are useful to select deep intronic variants that are likely to be worth RNA analysis. However, extensive in silico analysis should be compared with transcript analysis results to determine their benefit in the context of molecular diagnosis.

In summary, two novel pathogenic *COL4A4* variants and four novel pathogenic *COL4A5* splicing variants were detected in four unrelated AS patients with negative NGS test results. All identified variants were deep intronic variants. As obtaining urine is feasible and non-invasive, we suggest analyzing *COL4A3–5* mRNA from urine as the preferred method for evaluation of patients with clinically diagnosed or suspected AS with negative results of NGS analysis of coding regions.

## Materials and methods

All methods were carried out in accordance with relevant guidelines and regulations.

### Ethical considerations

The Ethical Committee of Peking University First Hospital approved the procedures in this study.

### Patients

Patients with hematuria or hematuria and proteinuria were enrolled from August 2019 to August 2020 by pediatric nephrologists from the Department of Pediatrics, Peking University First Hospital based on fulfillment of the following two criteria: diagnosed or suspected AS and no pathogenic *COL4A3–5* variants identified by exome sequencing. Patients were diagnosed with AS if they met one of the following three criteria: 1. abnormal staining of the type IV collagen α5 chain in skin and/or renal specimens; 2. ultrastructural alterations in the GBM typical of AS; or 3. positive family history of hematuria and/or ESRD. Informed consent was obtained from adult subjects or the parents or legal guardians of the subjects who were less than 18 years of age. Patients were excluded if they were unwilling to participate in the study.

### Urine mRNA extraction, and sequence of NPHS2 and COL4A3-5 cDNA of the control

**Figure 6:**
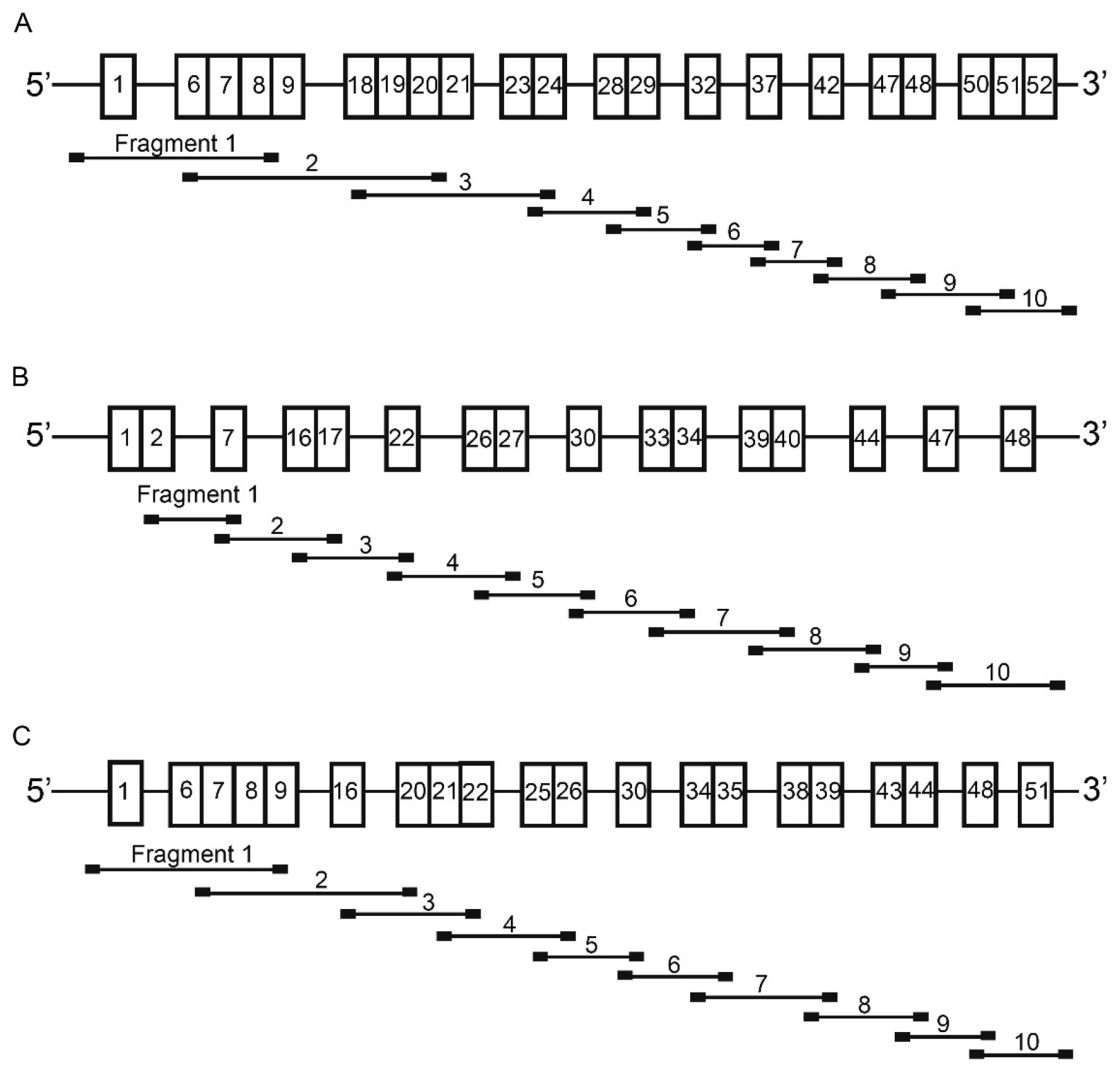
Strategy for amplification of the *COL4A3-5* cDNAs by PCR. (**A**) The entire coding region of *COL4A3* mRNA was demonstrated by all boxes. (**B**) The entire coding region of *COL4A4* mRNA was showed by all boxes. (**C**) The entire coding region of *COL4A5* mRNA was indicated by all boxes. The exon numbers of each gene were represented by the numbers in the boxes. The localizations of the ten amplified fragments were demonstrated with the primer site (black squares).

To obtain fresh urine, a healthy volunteer without hematuria or proteinuria was asked to drink approximately 1000–1500 ml water rapidly after emptying the bladder and spontaneously void every 30–45 min. Approximately 500 ml of urine per patient was collected and allocated in 50 mL centrifuge tubes pre-treated with RNAlater (Qiagene, 145,023,696). Urine samples were centrifuged for 5 min (1200 rpm at 4 °C), and the supernatants were carefully removed using pipettes. The urinary pellets were washed twice with ice-cold PBS supplemented with RNAlater (1 ml RNAlater per 50 ml PBS) and the samples were centrifuged for 5 min (1200 rpm at 4 °C). Total RNA was isolated from urinary pellets using TRIzol reagent (Gibco, Grand Island, NY, USA) according to the manufacturer’s instructions. The concentration of RNA was quantified with a NanoDrop 2000 spectrophotometer (Thermo Fisher Scientific, Waltham, MA, USA). Reverse-transcription was performed using the RevertAid First Strand cDNA Synthesis Kit (TAKARA, K1622). A podocyte protein podocin is encoded by *NPHS2* gene, so this gene was used to assess the podocytes in urine. A 388 bp fragment of *NPHS2* (NM_014625.3) cDNA, including exons 3, 4, 5 and partial sequences of exons 2 and 6, was amplified by PCR using a pair of primers (F: 5’- GGTACCAAATCCTCCGGCTTA-3’, R: 5’- CCAAGGCAACCTTTGCATCTT -3’). Ten pairs of PCR primers were designed to amplify the entire coding sequence of COL4A3 (NM_000091.5), COL4A4 (NM_000092.5), and COL4A5 (NM_000495.5), respectively; the sequences were listed in Table 3. Figure 6 showed the strategy for amplification of the *COL4A3-5* cDNAs by PCR. The ‘Touchdown’ PCR procedure included annealing from 64 °C to 57 °C, descending 1 °C every two cycles, followed by annealing at 57 °C for 26 cycles. The PCR amplification products were checked by 2% agarose gel electrophoresis and sequenced on an ABI 3730XL (SinoGenoMax Company Limited, China).

**Table 3:** Primers for *COL4A3*-*5* cDNA analysis.

Fragment	Forward prime (5ʹ–3ʹ)	Reward prime (3ʹ–5ʹ)	Product (bp)
*COL4A3*-1	GCGAGGCGAGCTTTCCAG	GGAGCACCCTTTTGTCCTTT	586
*COL4A3*-2	TACGGACTTGTCGGTGTACC	GGCCTTGATGATCCAGGACT	815
*COL4A3*-3	GGGTGAAGATGGCATTAAGGG	AGACCTGTATTTCCTGGGGAC	594
*COL4A3*-4	CAGGAAGACAAGGCGCAG	GACAACCCAGTGATCCTTTTGT	666
*COL4A3*-5	CTGGGGAAATGTGGAGATCC	TGGCCCTAAAATTCCCGGAT	618
*COL4A3*-6	CACTGGGTCAAAGAGGATATCC	GTCCTGGCCTTGTACCTTCT	591
*COL4A3*-7	CAGGGAGATAAGGGAGAGCC	GCTTCCTCTTGAGCCTGGT	600
*COL4A3*-8	GTGCAATTATCCCTGGCCAG	CCTTTCAAACCTGGCAATCC	598
*COL4A3*-9	GGCAAGGATGGAAAACCAGG	GCACGTTCCTCTTCCATGAC	687
*COL4A3*-10	CGGCTGGATTTCTCTCTGGA	ACAGCACAGATTAGAGACCCA	498
*COL4A4*-1	TGACCCAGAACACAGAACCT	CCAAGAGCTCCTCTTCCTCC	564
*COL4A4*-2	CCACAATGGCTCAAGAGGTG	TCCAAATAGCCCAGGATCTCC	595
*COL4A4*-3	TCCTGGTTCCTATGGATCTCC	ACCTTTTGTTCCAAGCCAGC	646
*COL4A4*-4	CTGTGAGCCTGGACCCATG	ATTTCCGCTGTTCCTGGTGT	672
*COL4A4*-5	CAACGTAACCTACCCTGGGA	CCAGGTAGCCCATCATCTCC	696
*COL4A4*-6	CAGCTGGAATGAAAGGCCTC	AGGTCCTCTTGCTCCATCAA	617
*COL4A4*-7	GCTCAACTGGTCTAAGAGGGT	GCTCTTCCTGTGGCACCT	652
*COL4A4*-8	GGGCTAAAAGGGGAGAGAGG	CTCTCATTCCAGGGAGCCC	583
*COL4A4*-9	CAGGCATGAGAGGACCAGAA	CTGACATAGGGGCGGATCG	538
*COL4A4*-10	TGCCCAGAGAAACGACAGAT	ACGTGTTGGTGAATTTCGCA	499
*COL4A5*-1	GCTCTCTCCATATAAACCCTCAA	TCCTGGCAGTGATGACATAATT	600
*COL4A5*-2	TGCAATGGAACCAAGGGAGA	TCATCACCTTTCTGACCCCTT	863
*COL4A5*-3	GCAAAGATGGAGAAAATGGCC	TGGAGGCCCTGAAATACCA	579
*COL4A5*-4	AGGAGAACAAGGAGTGAAAGGT	CCGGCTGGGTTATAGTCTGA	584
*COL4A5*-5	CATACAAGGTGTGGCAGGAA	CTATTGGCCCAGGAATCCC	596
*COL4A5*-6	GCCAGGAATAGGTGTTCAGG	CGATGGTTCCTTTAAGTCCAGG	644
*COL4A5*-7	ACCTGGACTGAGTGGACAAC	GTTCACCCTTCTGTCCAGCT	575
*COL4A*5-8	TGTAGGTGGTGGAGGTCATC	ACCAATAAGTCCCGGTTCCC	613
*COL4A5*-9	GGATTCCCAGGCATGAAAGG	CCTTTAGGGGTTGCATGCTC	673
*COL4A5*-10	GCCTTTCATGTTCTGCAAC	GGGGACAATGAGACACTGCA	594

The reference sequence of *COL4A3-5* transcripts was NM_000091.5, NM_000092.5, and NM_000495.5, respectively.

### Analysis of urine COL4A3–5 mRNA from AS patients

For AS patients with an uncertain inheritance pattern, *COL4A3–5* mRNAs from urine were analyzed. When available, RNA from parents was sequenced to assess the segregation of variants with the disease in the respective families. RT-PCR and direct sequencing followed the above protocol.

### Analysis of COL4A5 mRNA from skin fibroblasts

For patients with XLAS, *COL4A5* mRNA from cultured skin fibroblasts was analyzed. Dermal fibroblasts were cultured as described previously^11^. Primers for *COL4A5* cDNA analyses were performed using the same primers as shown in Table 3. RT-PCR and direct sequencing followed the above protocol.

### Genomic DNA analysis

Genomic DNA was extracted from peripheral blood lymphocytes. Once abnormal *COL4A3–5* transcripts were detected, the corresponding exons with flanking intronic sequences were further analyzed using PCR and direct sequencing to identify the point variants that may cause new splice sites. PCR primers are available on request. The public database gnomAD (http://gnomad-sg.org/) was used to assess the frequencies of variants. HGMD and ClinVar were used to detect previously reported pathogenic variants.

### Haplotype analysis

According to the report of Tazón-Vega et al^13^, three short tandem repeats (DXS1120, DXS6802, and DXS1210) around the *COL4A5* gene were used to perform linkage analysis in II-4 (proband 2), II-5 and III-1 of family 2. PCRs were performed using the published primers (the forward primer in each pair was labeled with 6-carboxyfluorescein fluorescence)^13^ and following the above protocol. PCR products were separated on a 3730XL automatic sequencer (Applied Biosystems) and analyzed by GeneMapper software (version 4.0).

### In silico splice tools for identifying deep intronic pathogenic variants

To evaluate the reliability of in silico splicing prediction tools in discriminating the deep intronic pathogenic variants identified in this study, five tools including HSF (http://www.umd.be/HSF/), NNSPLICE (http: //www.fruitfly.org/seq_tools/splice.html), NetGene2 (http://www.cbs.dtu.dk/services/NetGene2/), SpliceAI (https://spliceailookup.broadinstitute.org/), and MaxEntScan (http://genes.mit.edu/burgelab/maxent/Xmaxentscan_scoreseq.html) were used. NNSPLICE and NetGene2 present scores of 0–1 for the predicted site; the higher the score the more likely a variant is a splicing site.

## Supplementary Information

Supplementary Information 1.

Supplementary Information 2.

Supplementary Information 3.

Supplementary Information 4.

Supplementary Information 5.

Supplementary Information 6.
