# Author Correction: mRNA analysis identifies deep intronic variants causing Alport syndrome and overcomes the problem of negative results of exome sequencing

**DOI:** 10.1038/s41598-021-01604-9

**Published:** 2021-11-09

**Authors:** Xiaoyuan Wang, Yanqin Zhang, Jie Ding, Fang Wang

**Affiliations:** Department of Pediatrics, Peking University First Hospital, Beijing, 100034 China

Correction to: *Scientific Reports* https://doi.org/10.1038/s41598-021-97414-0, published online 10 September 2021

The original version of this Article contained errors in the mRNA sequences.

As a result, in the Results section, under subheading ‘Analysis of urine NPHS2 and COL4A3‑5 mRNAs of the control’,

“The sizes of all ten overlapping fragments covering the entire coding sequence of either *COL4A3*, *COL4A4*, or *COL4A5* mRNA were the same as originally conceived (Fig. 1C), and sequencing of these RT–PCR products confirmed that the amplified sequences mapped precisely to the published *COL4A3*, *COL4A4*, and *COL4A5* mRNA sequences (NM_000091.5, NM_000092.5, and NM_000495.5), respectively.”

now reads:

“The sizes of all ten overlapping fragments covering the entire coding sequence of either *COL4A3*, *COL4A4*, or *COL4A5* mRNA were the same as originally conceived (Fig. 1C), and sequencing of these RT–PCR products confirmed that the amplified sequences mapped precisely to the published *COL4A3*, *COL4A4*, and *COL4A5* mRNA sequences (NM_000091.5, NM_000092.5, NM_000495.5, and NM_033381), respectively.”

Additionally, Table 1 legend contained an error in the number of probands.

“*Age at which serum creatinine tests were carried out in the three probands was 25, 30, 10 and 14 years, respectively.”

now reads:

“*Age at which serum creatinine tests were carried out in the four probands was 25, 30, 10 and 14 years, respectively.”

Finally, in the Discussion section, the following sentence was erroneously included and has subsequently been removed.

“Genetic linkage analysis demonstrated co-segregation of the disease. the proband shared the haplotype of three microsatellite markers around the *COL4A5* gene with her affected daughter, analysis showed that the same haplotype was carried by all affected males and obligatory carrier females.”

The original Article has been corrected.

