# Supplementary figures and images for: mRNA analysis identifies deep intronic variants causing Alport syndrome and overcomes the problem of negative results of exome sequencing
Source: Sci Rep. 2021 Sep 10;11:18097. doi: 10.1038/s41598-021-97414-0 (PMC8433132; doi:10.1038/s41598-021-97414-0)

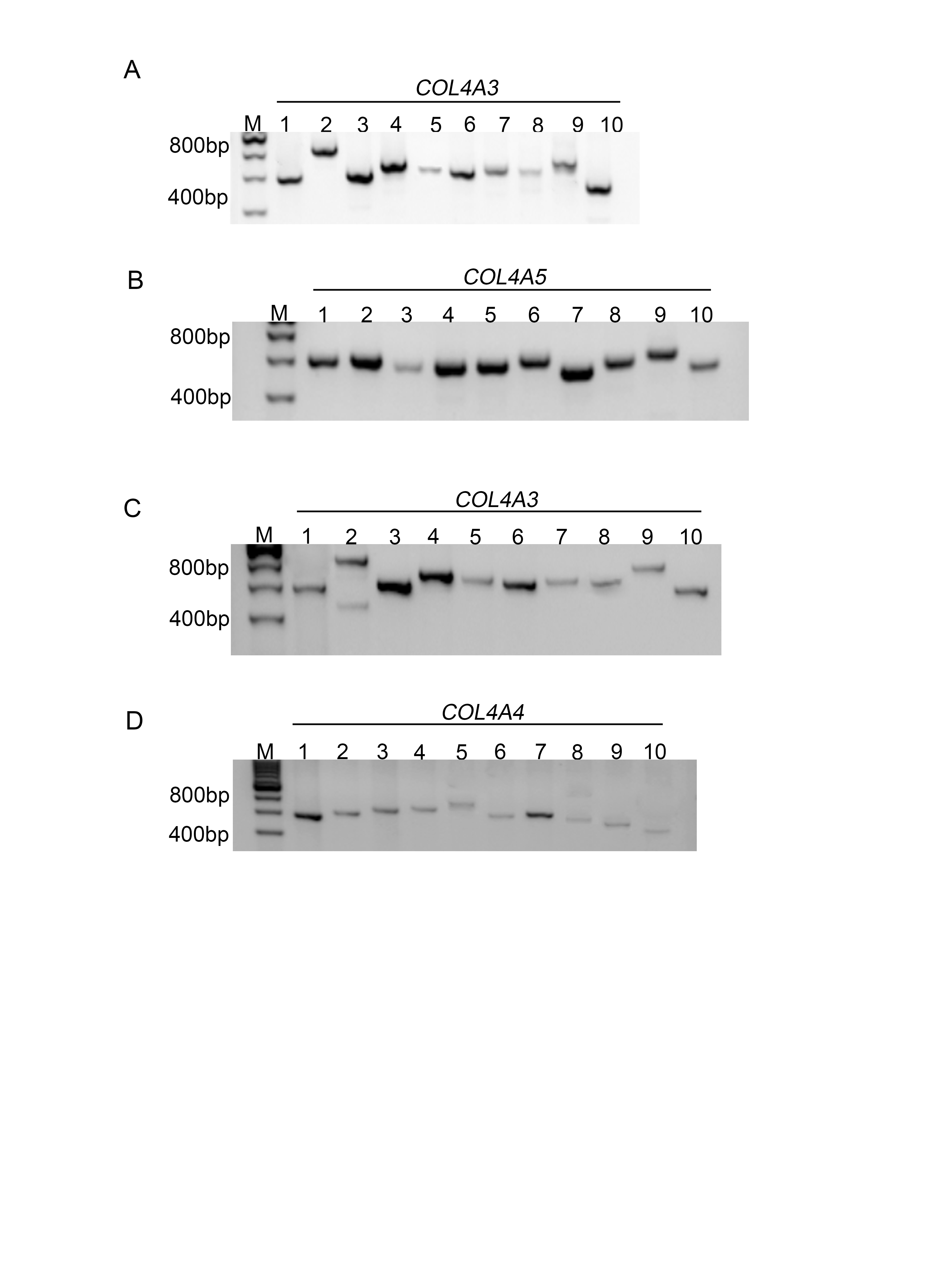

Supplement: Supplementary file 1 — Supplementary Information 1. [file 41598_2021_97414_MOESM1_ESM.jpg]

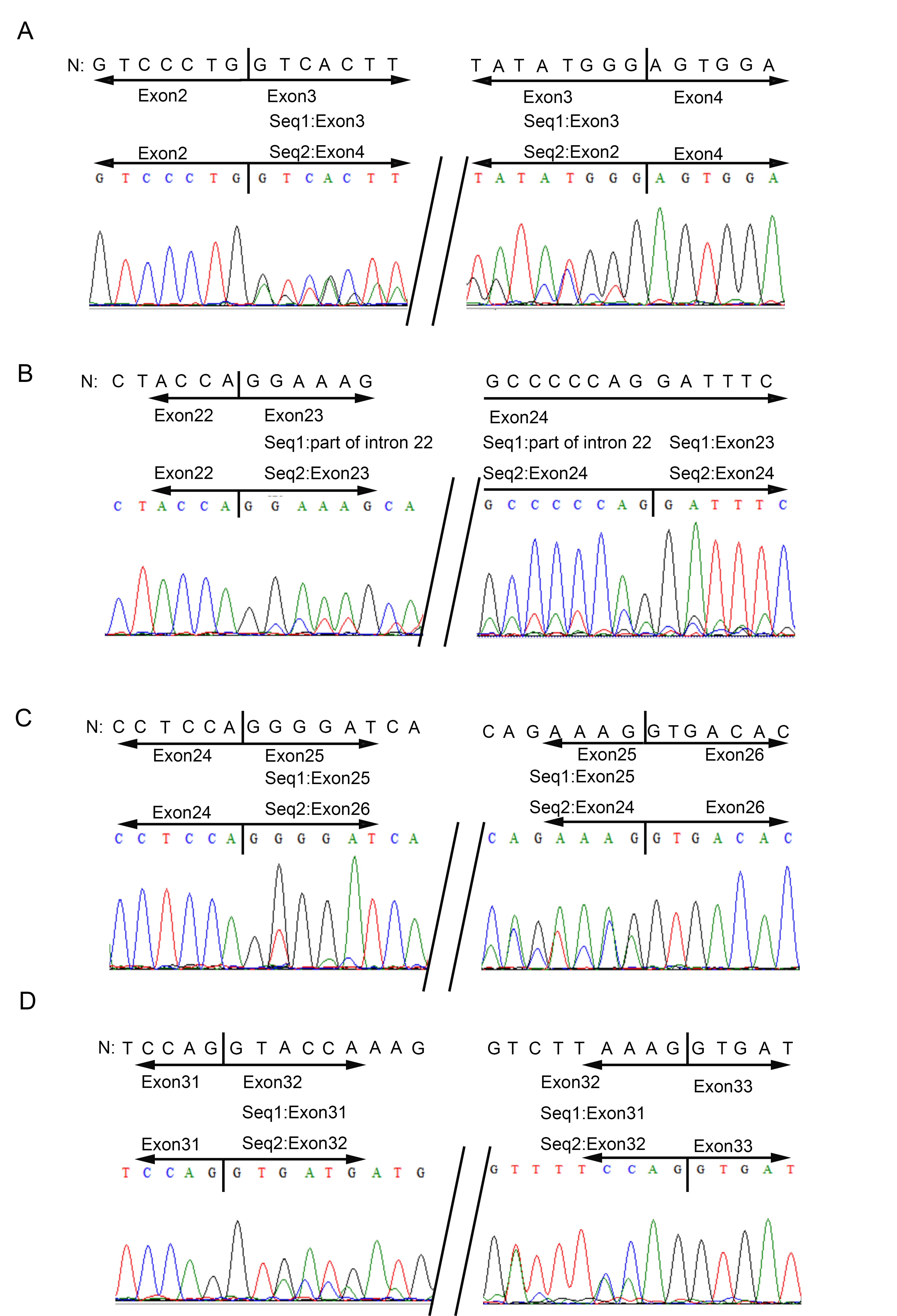

Supplement: Supplementary file 2 — Supplementary Information 2. [file 41598_2021_97414_MOESM2_ESM.jpg]

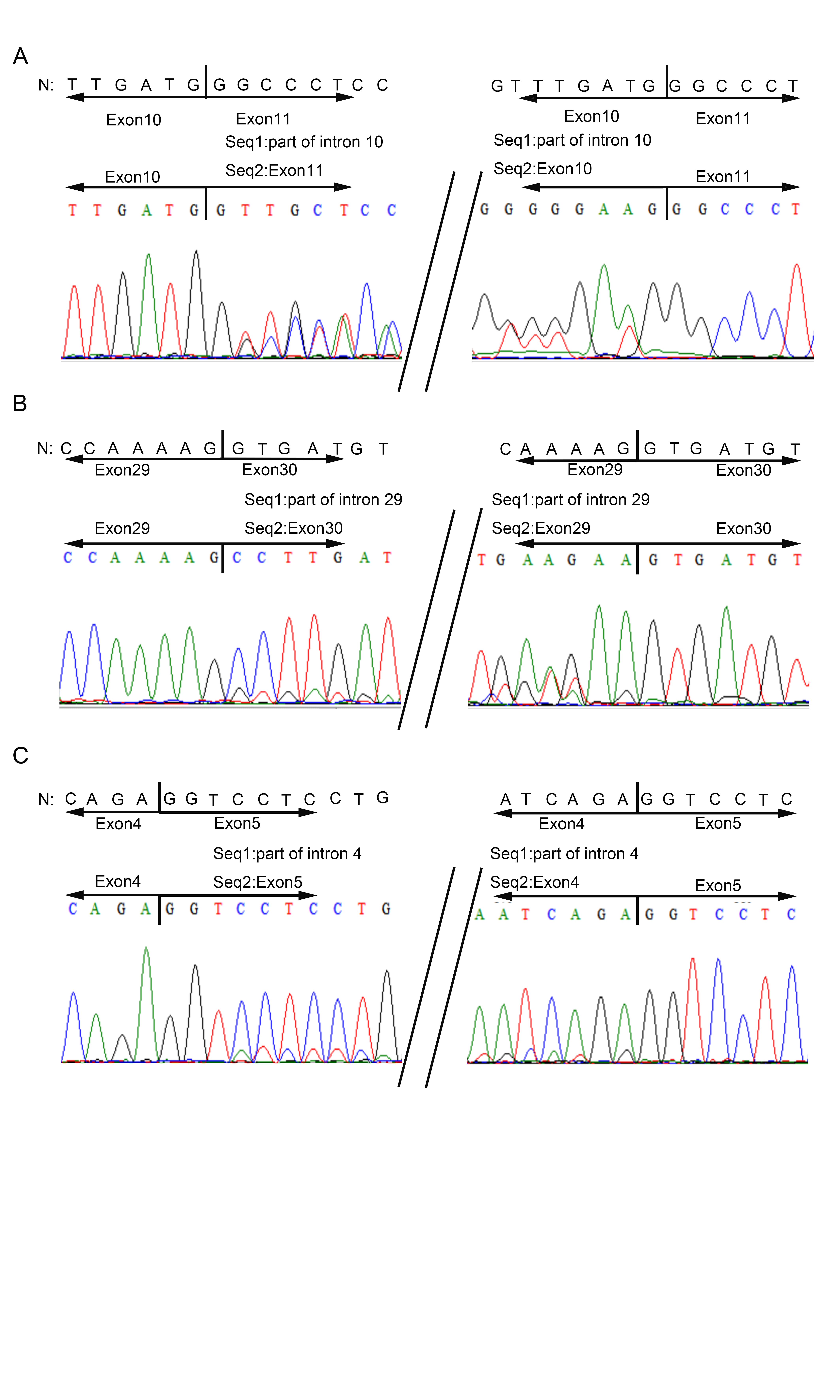

Supplement: Supplementary file 3 — Supplementary Information 3. [file 41598_2021_97414_MOESM3_ESM.jpg]

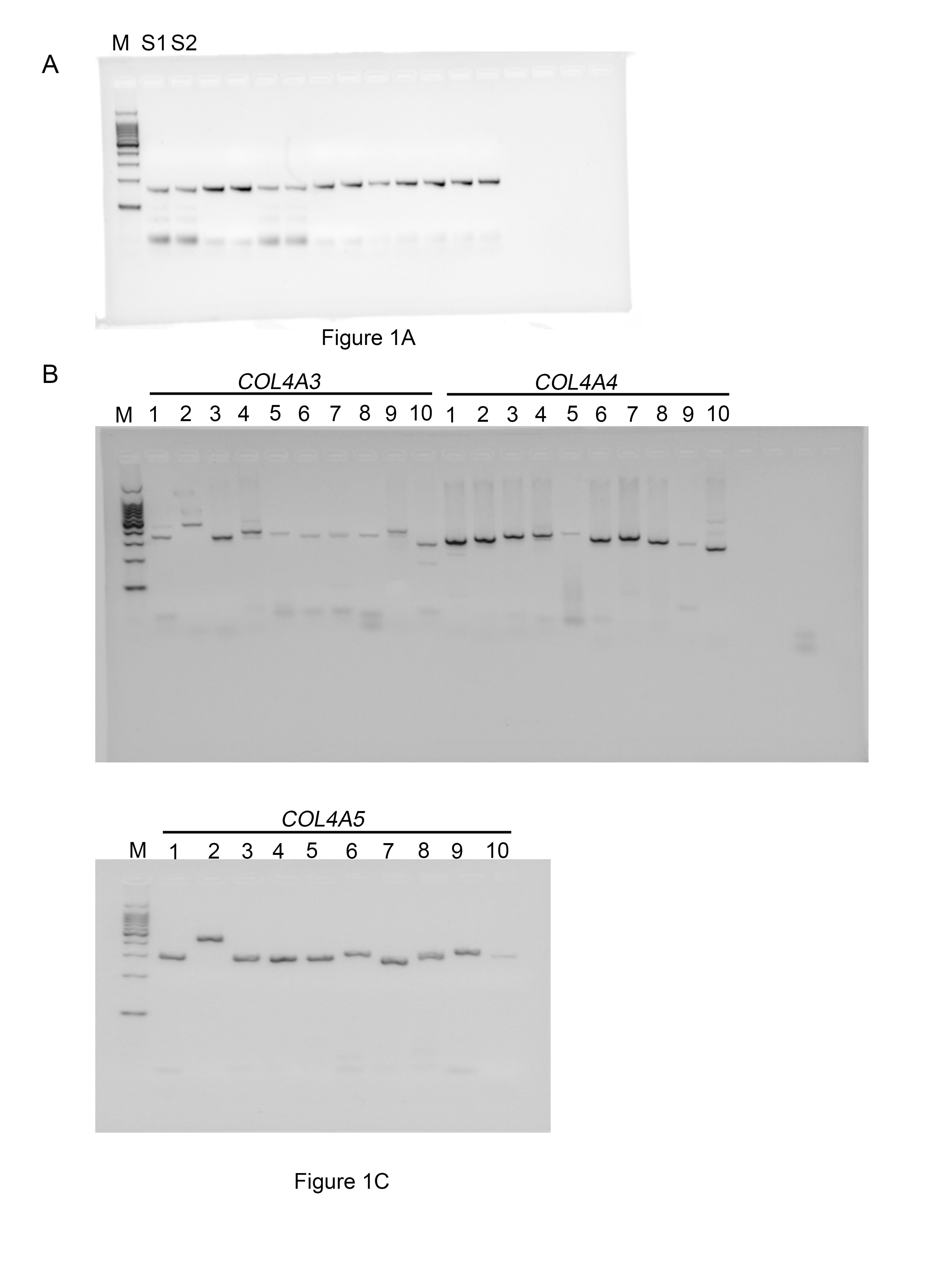

Supplement: Supplementary file 4 — Supplementary Information 4. [file 41598_2021_97414_MOESM4_ESM.jpg]

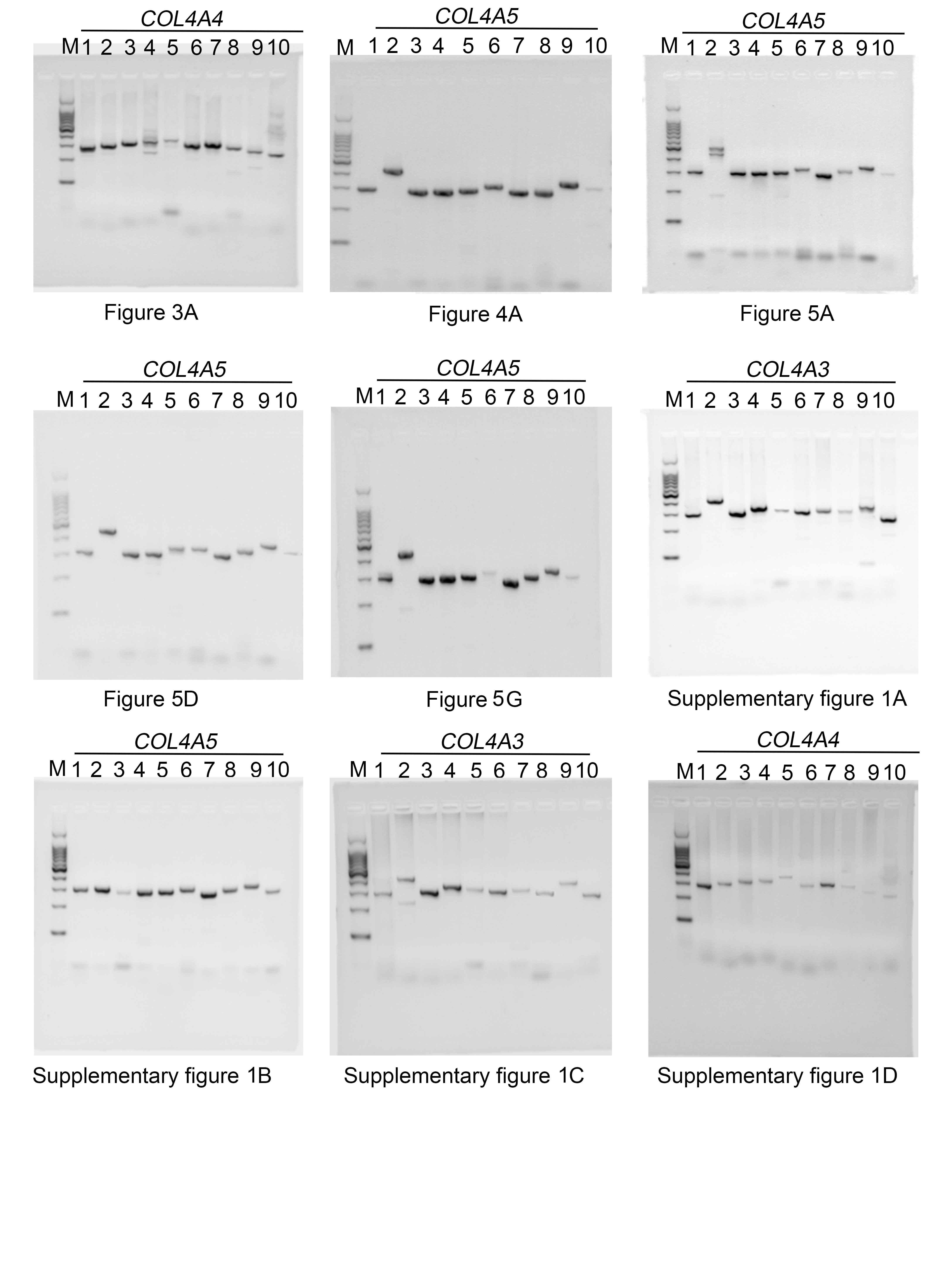

Supplement: Supplementary file 5 — Supplementary Information 5. [file 41598_2021_97414_MOESM5_ESM.jpg]
