# Supplementary material for: mRNA analysis identifies deep intronic variants causing Alport syndrome and overcomes the problem of negative results of exome sequencing
Source: Sci Rep. 2021 Sep 10;11:18097. doi: 10.1038/s41598-021-97414-0 (PMC8433132; doi:10.1038/s41598-021-97414-0)
Supplement: Supplementary file 6 — Supplementary Information 6. [file 41598_2021_97414_MOESM6_ESM.docx]

Supplementary Figure 1. Agarose gel images of RT-PCR products for probands 1 and 2 urine.

M: DNA molecular mass marker. Lane 1 to 10: 10 overlapping PCR products covering the entire targeted gene cDNA.

Supplementary Figure 2. Sequencing of RT-PCR products containing the abnormal transcripts from urine.

A-C: proband 1. D: proband 2. The junctions of exons or exons and introns are shown by the vertical line, respectively. N: normal sequence. Seq: sequence.

Supplementary Figure 3. Sequencing of RT-PCR products containing the abnormal transcripts from skin fibroblasts.

A: proband 3. B: proband 4. C: proband 5. The junctions of exons or exons and introns are shown by the vertical line, respectively. N: normal sequence. Seq: sequence.

Supplementary Figure 4. The original image of Figure 1A and figure 1C.

Supplementary Figure 5. The original image of Figure 3A, 4A, 5A, 5D, 5G, and Supplementary Figure 1.
